# Clinical correlation between platelet parameters and coronary artery lesions in pediatric Kawasaki disease

**DOI:** 10.3389/fped.2026.1858138

**Published:** 2026-06-09

**Authors:** Yujia Ji, Xia Chen, Lizhi Jiang, Chao Li

**Affiliations:** 1 Pediatrics, Shiyan Maternal and Child Health Hospital, Shiyan, China; 2 Pediatric Ward 3, Taihe Hospital, Shiyan, China; 3 Department of Neurology, Taihe Hospital, Hubei University of Medicine, Hubei, China

**Keywords:** coronary artery lesions, correlation, Kawasaki disease, platelet, propensity score matching

## Abstract

**Objective:**

To investigate the clinical correlation between the occurrence of coronary artery lesions and platelet levels in children with Kawasaki disease.

**Methods:**

Propensity score matching was used to compare platelet parameters (PLT, MPV, PCT, PDW) between KD children and healthy controls. KD patients were divided into lesion and non-lesion groups according to coronary artery lesions. Harada score and multiple linear regression were applied to assess risk and correlation.

**Results:**

No significant differences were observed in baseline data between the two groups (*P* > 0.05), indicating comparability. Platelet parameters (PLT, MPV, PCT, PDW) in the experimental group were significantly higher than those in the control group (319.56 ± 26.17 vs. 201.56 ± 12.98; 8.94 ± 1.25 vs. 7.05 ± 1.13; 0.30 ± 0.08 vs. 0.13 ± 0.02; 20.75 ± 2.10 vs. 16.17 ± 1.89, all *P* < 0.05). The lesion group showed significantly higher platelet parameters than the non-lesion group (330.56 ± 31.89 vs. 303.89 ± 22.73; 9.41 ± 1.35 vs. 8.32 ± 1.19; 0.36 ± 0.06 vs. 0.24 ± 0.09; 22.47 ± 1.88 vs. 19.18 ± 1.76, all *P* < 0.05). The Harada score was significantly higher in the lesion group than in the non-lesion group (5.82 ± 1.39 vs. 4.17 ± 1.76, *P* < 0.05). Multivariate binary logistic regression analysis showed significant correlations between PLT, MPV, PCT, PDW and coronary artery lesions (*P* < 0.05).

**Conclusion:**

The occurrence of coronary artery lesions in children with Kawasaki disease is closely associated with platelet levels. These findings suggest that platelet parameters are associated with coronary artery lesions in children with Kawasaki disease. Further prospective studies are needed to validate their clinical utility in risk stratification and management.

## 1 Introduction

Kawasaki Disease (KD) is an acute self-limiting disease characterized by systemic vasculitis, predominantly affecting children under 5 years of age. Its incidence has been steadily increasing annually, making it one of the leading causes of acquired heart disease in children in certain countries and regions (1–3). According to the White Paper on the Current Status of Kawasaki Disease Diagnosis and Treatment in China, the annual incidence of KD in China ranges from approximately 0.87‰–1.32‰, with notable regional variations and seasonal clustering. The most severe complication of KD is coronary artery lesions (CAL), including coronary artery dilation, aneurysm formation, and even thrombotic occlusion. The incidence of CAL can reach 15%–25% in children who do not receive timely treatment, with approximately 2% of cases progressing to ischemic heart disease or sudden cardiac death, posing a significant long-term threat to children's cardiovascular health.

Platelet activation plays a central role in the vasculitis process of KD. Pathological studies have demonstrated that during the acute phase of KD, there is a synergistic effect of excessive platelet activation, endothelial cell injury, and cytokine storm, leading to vascular endothelial dysfunction and the formation of a prothrombotic state (4, 5). However, existing research has primarily focused on the association between platelet parameters and the overall severity of KD, with limited systematic validation of their direct correlation with the specific complication of coronary artery lesions. The Harada scoring system, an important tool for assessing the severity of KD, integrates multidimensional indicators such as duration of fever, C-reactive protein (CRP) levels, and platelet count, and its clinical value has been widely recognized (6, 7). Based on the above, this study conducted a retrospective analysis of clinical data from 80 children with KD admitted to our hospital from August 2023 to December 2024. Children were further divided into a lesion group and a non-lesion group based on the occurrence of coronary artery lesions. By combining the Harada scoring system and multiple linear regression analysis, this study systematically explored the association between four platelet parameters (PLT, MPV, PCT, PDW) and coronary artery lesions, aiming to provide may serve as candidate biomarkers for the clinical management of KD.

## 2 Materials and methods

### 2.1 Study subjects

After rigorous screening based on inclusion criteria, this study selected 80 children with Kawasaki Disease (KD) admitted to our hospital from August 2023 to December 2024 as the experimental group to ensure sample representativeness. Additionally, 80 healthy children undergoing physical examinations at our hospital during the same period were included as the control group. The control group was included to provide reference values for platelet parameters, but the primary analysis focused on comparisons within the KD cohort. This study was designed as a retrospective investigation, with the research protocol approved by the Ethics Committee of our hospital and conducted in compliance with the Declaration of Helsinki. Given the retrospective nature of this study and the use of pre-existing clinical data, the Ethics Committee waived the requirement for patient informed consent. Privacy safeguards for all case data were implemented through the following measures: anonymization prior to data collection, with removal of personally identifiable information (e.g., names, ID numbers); establishment of a tiered access control system restricting full dataset access to authorized researchers only; and presentation of clinical data in aggregated form during publication to eliminate risks of individual information disclosure. This study was an exploratory, retrospective analysis. No *a priori* sample size calculation was performed; all eligible KD patients admitted during the study period were included.

### 2.2 Inclusion and exclusion criteria

Inclusion criteria required compliance with the diagnostic criteria outlined in the Diagnostic Guidelines for Kawasaki Disease (8, 9); clinical manifestations must include at least two of the following features: hard swelling of the hands and feet, skin desquamation or erythema, non-suppurative lymphadenopathy, dry and red lips with fissures, diffuse hyperemia of the pharyngeal mucosa, bilateral conjunctival injection, and rash; additionally, complete clinical data, fever duration exceeding 3 days, and absence of concurrent immune system or hematologic disorders were required.

Exclusion criteria included: history of congenital heart disease or inherited metabolic disorders; recent use of antiplatelet agents; presence of infectious, allergic, or chronic diseases; hematologic disorders; corticosteroid treatment within the past 3 months; or incomplete clinical data.

### 2.3 Grouping and data collection

CAL was defined as a coronary artery *Z*-score ≥ 2.0 (adjusted for body surface area) or the presence of an aneurysm, according to the 2017 AHA guidelines. Echocardiography was performed within 24–48 h of admission (pre-IVIG) and repeated at 2–4 weeks post-IVIG. All studies were interpreted by a single blinded pediatric cardiologist. Based on echocardiographic findings, the study group was divided into a lesion subgroup and a non-lesion subgroup. The lesion subgroup comprised children with coronary artery dilation or aneurysmal changes, while the non-lesion subgroup included those with normal coronary artery morphology.

Data collection involved two components: recording of basic demographic characteristics and measurement of blood parameters. First, baseline information—including gender distribution, age range, and fever duration—was systematically documented for both groups. Venous blood samples were collected within 24 h of admission and prior to the initiation of intravenous immunoglobulin (IVIG) or aspirin therapy. All patients were in the acute phase of Kawasaki disease (fever duration ≥ 3 days) at the time of sampling. And platelet parameters were measured using a DxH800 automated hematology analyzer (Beckman Coulter), including platelet count (PLT), mean platelet volume (MPV), platelet hematocrit (PCT), and platelet distribution width (PDW). Disease severity was assessed using the Harada risk scoring system (maximum score: 7), with scores ≥4 indicating high-risk status requiring close monitoring of disease progression.

### 2.4 Statistical methods

Data analysis was performed using SPSS 26.0 software. Continuous variables are presented as mean ± standard deviation (*x¯* ± *s*), with intergroup comparisons conducted via *t*-tests. Categorical variables are expressed as frequencies (%), with intergroup comparisons performed using chi-square tests. Multivariate logistic regression analysis was used to identify independent predictors of coronary artery lesions, adjusting for age, sex, fever duration, IVIG timing (within 10 vs. >10 days), CRP level, incomplete Kawasaki disease status, and treatment response. Results were presented as odds ratios (OR) and 95% confidence intervals (CI). Statistical significance was defined as *P* < 0.05. Model assumptions (linearity of logit, multicollinearity, goodness-of-fit) were checked and satisfied. Statistical significance was defined as *P* < 0.05.

## 3 Results

### 3.1 Baseline data

At baseline, there were no significant differences in age or gender distribution between the experimental and control groups (*P* > 0.05, Table 1).

**Table 1:** Comparison of baseline data between two groups.

		Experimental group	Control group	*t*	*P*
Number of cases	–	80	80	–	–
Gender	Male	46	48	–	–
	Female	34	32	0.442	0.624
Age (years)	–	0.5–10	0.5–10	–	–
	Mean	6.11 ± 2.39	6.17 ± 2.25	0.458	0.612

### 3.2 Platelet parameters

#### 3.2.1 Experimental group vs. control group

**Figure 1:**
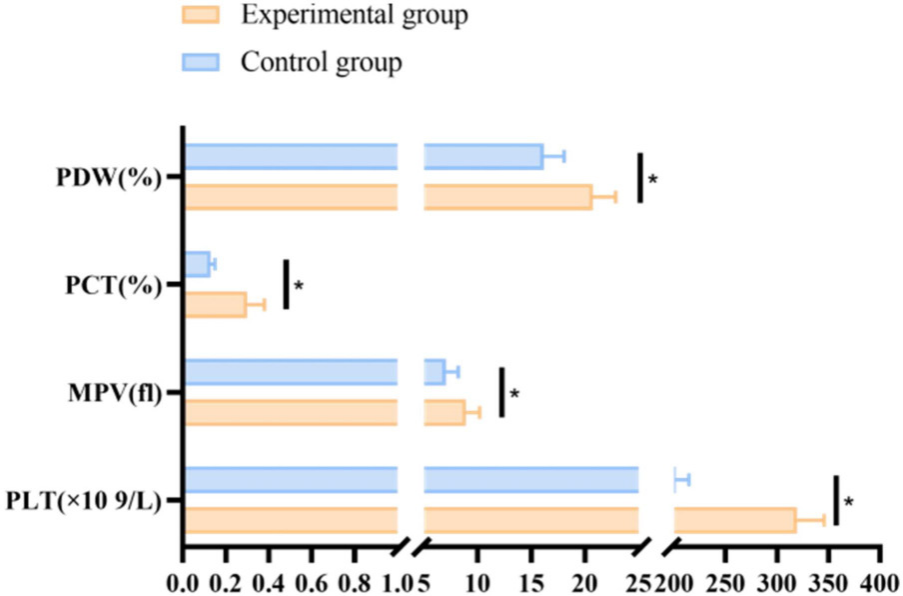
Comparison of platelet parameters between experimental and control groups. *Indicates a significant difference between groups (*P* < 0.05).

The levels of four platelet parameters (PLT, MPV, PCT, PDW) in the experimental group were significantly higher than those in the control group (319.56 ± 26.17/8.94 ± 1.25/0.30 ± 0.08/20.75 ± 2.10 vs. 201.56 ± 12.98/7.05 ± 1.13/0.13 ± 0.02/1.16.17 ± 1.89; *P* < 0.05) (see Figure 1).

#### 3.2.2 Lesion subgroup vs. non-lesion subgroup

**Figure 2:**
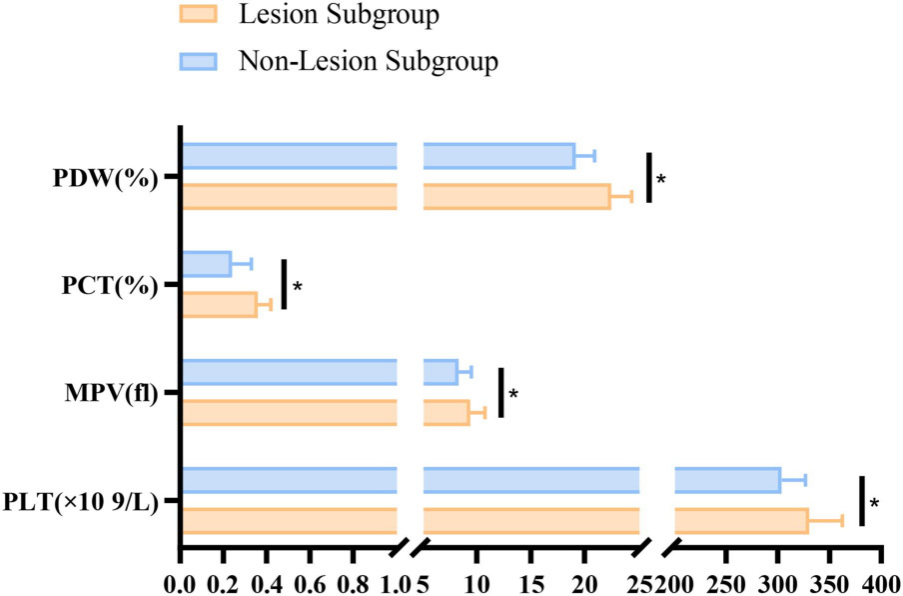
Comparison of platelet parameters between lesion and non-lesion groups. *Indicates a significant difference between groups (*P* < 0.05).

All four platelet parameters were significantly higher in the lesion subgroup than in the non-lesion subgroup (*P* < 0.05; detailed in Figure 2), showing that all four platelet parameters (PLT, MPV, PCT, and PDW) were significantly independently associated with the presence of coronary artery lesions.

### 3.3 Lesion severity

**Figure 3:**
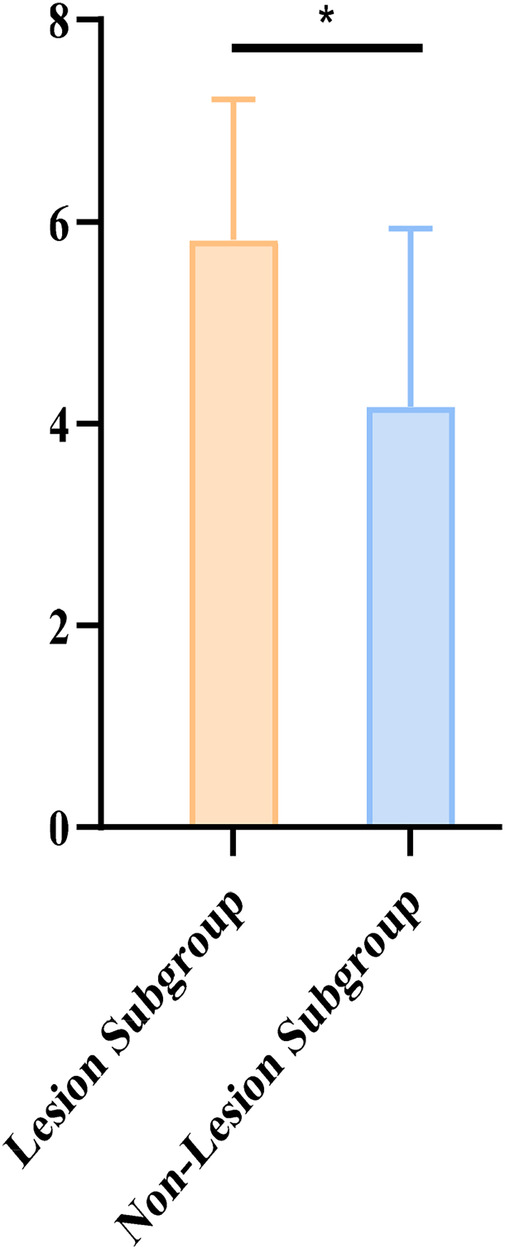
Comparison of Harada scores between lesion and non-lesion subgroups. *Indicates a significant difference between groups (*P* < 0.05).

The Harada score in the lesion subgroup was significantly higher than that in the non-lesion subgroup (5.82 ± 1.39 vs. 4.17 ± 1.76; *P* < 0.05) (see Figure 3).

### 3.4 Correlation analysis

To identify independent predictors of coronary artery lesions (CAL), we performed multivariate binary logistic regression analysis, adjusting for age, sex, fever duration, IVIG timing, CRP level, incomplete KD status, and treatment response. As shown in Table 2, all four platelet parameters (PLT, MPV, PCT, and PDW) remained independent predictors of CAL after adjusting for these covariates (all *P* < 0.05). Specifically, PLT showed the strongest association (OR = 1.15, 95% CI: 1.04–1.27, *P* = 0.011), followed by PDW (OR = 1.45, 95% CI: 1.08–1.95, *P* = 0.015), PCT (OR = 1.32, 95% CI: 1.05–1.66, *P* = 0.019), and MPV (OR = 1.28, 95% CI: 1.04–1.58, *P* = 0.021) (see Table 2).

**Table 2:** Multivariate binary logistic regression analysis of platelet parameters as predictors of coronary artery lesions.

Variable	Odds ratio (OR)	95% CI	*P* value
PLT	1.15	1.04–1.27	0.011
MPV	1.28	1.04–1.58	0.021
PCT	1.32	1.05–1.66	0.019
PDW	1.45	1.08–1.95	0.015

## 4 Discussion

Platelet parameters have been shown to reflect disease activity (4, 10, 11). This study systematically analyzed their correlation with coronary artery lesions (CAL) in Kawasaki disease (KD).

The baseline demographic characteristics were comparable between the two groups. The phenomenon that the four platelet parameters (PLT, MPV, PCT, PDW) in the experimental group were significantly higher than those in the healthy control group (*P* < 0.05) requires in-depth interpretation from the pathophysiological perspective of KD. Elevated PLT may reflect compensatory bone marrow hyperplasia or reduced peripheral platelet consumption, aligning with the mechanism of increased platelet production induced by the cytokine storm during the acute phase of KD. Increased MPV suggests larger platelet size and enhanced activity, likely resulting from abnormal differentiation of megakaryocytes stimulated by inflammatory mediators. PCT, as the product of PLT and MPV, directly reflects increased platelet mass, which correlates positively with disease activity. Elevated PDW indicates increasedheterogeneity in platelet volume, possibly due to metabolic abnormalities caused by excessive platelet activation or compensatory bone marrow dysfunction (12–15). These parameter changes collectively form a biomarker profile for KD vasculitis, and their dynamic monitoring can provide quantitative evidence for disease staging and therapeutic efficacy evaluation.

The finding that platelet parameters were significantly higher in the lesion subgroup than in the non-lesion subgroup (*P* < 0.05) reveals a direct association between platelet activation and coronary artery lesions. Coronary artery dilation or aneurysmal changes, the most severe complications of KD, have pathogenesis closely linked to platelet-mediated thrombosis, endothelial cell injury, and inflammatory cytokine infiltration. Specifically, elevated PLT may enhance platelet aggregation capacity by promoting thromboxane A2 secretion, while increased MPV may exacerbate vascular endothelial cell injury. Elevated PCT may directly reflect increased local thrombus burden in the coronary arteries, and elevated PDW may predict local hemodynamic disturbances caused by heterogeneous platelet function (16–18). This gradient pattern of platelet parameter changes provides early warning signals for identifying children at high risk of coronary artery lesions.

The result that the Harada score was significantly higher in the lesion subgroup than in the non-lesion subgroup (*P* < 0.05) further validates the dose-effect relationship between platelet parameters and disease severity. The Harada scoring system, a classic tool for assessing risk stratification in KD, includes indicators such as fever duration and CRP levels, which exhibit synergistic effects with platelet activation (19, 20). This study found that the probability of children being in a high-risk state significantly increased when the score was ≥4, forming a positive feedback loop with abnormally elevated platelet parameters (21–23). Notably, the platelet count indicator in the Harada score (which assigns a point for PLT < 350,000) correlates with the PLT findings in this study: among all KD patients, higher PLT values (within the range below 350,000) were associated with higher Harada scores and more severe coronary artery lesions. This consistency supports the use of a multidimensional evaluation system for disease prognosis.

Multivariate logistic regression analysis revealed that PLT, MPV, PCT, and PDW were all independent predictors of coronary artery lesions after adjusting for confounders (all *P* < 0.05). The odds ratios indicated a gradient effect: PLT showed the strongest association (OR = 1.15), followed by PDW (OR = 1.45), PCT (OR = 1.32), and MPV (OR = 1.28). This suggests that while each parameter contributes independently, PLT may have the most significant predictive efficacy for CAL, suggesting a potential role for its monitoring in clinical settings (24–27).

The results of this study need to be interpreted within a broader pathophysiological framework. The development of vasculitis in Kawasaki disease (KD) involves multiple mechanisms, including immune complex deposition, oxidative stress responses, and vascular endothelial cell apoptosis. Platelets, as important effector cells in inflammatory responses, not only influence thrombosis but also participate in vascular remodeling through the release of microparticles and the expression of adhesion molecules. The abnormal changes in platelet parameters observed in this study may be quantitative manifestations of these complex pathological processes. Additionally, the correlation between platelet parameters and the Harada score suggests a complementary relationship between traditional scoring systems and emerging biomarkers, and their combined application may optimize the precise diagnosis and treatment pathways for KD.

Although this study systematically explored the clinical correlation between coronary artery lesions and platelet levels in children with KD, several limitations cannot be overlooked. First, the study design was a single-center retrospective analysis, and sample selection may have been subject to selection bias. For example, the 80 children with KD included in the study may not fully represent the characteristics of the overall KD population, especially those from different regions and with varying levels of medical care, which may limit the external validity of the study results. Second, despite the use of propensity score matching to balance baseline data, information bias may still exist during retrospective data collection. For instance, the completeness and accuracy of clinical data records may be influenced by the quality of medical record documentation, particularly for subjective indicators such as fever duration and symptom descriptions. Third, this study only examined four platelet parameters (PLT, MPV, PCT, PDW) and did not include other relevant inflammatory factors (e.g., IL-6, TNF-α), vascular endothelial function indicators (e.g., vWF, ET-1), or coagulation function parameters, which may result in an incomplete interpretation of the pathological mechanisms of KD and hinder a deeper understanding of the interactions between platelet activation and other pathological processes. Furthermore, the study did not conduct long-term follow-up, making it impossible to assess the association between platelet parameters and the long-term prognosis of KD (e.g., the progression and recurrence risk of coronary artery lesions) or to dynamically observe the changes in platelet parameters during treatment and their relationship with therapeutic efficacy. Fifth, the timing of laboratory measurements was not fully standardized. Although all blood samples were collected within 24 h of admission (prior to IVIG and aspirin therapy) and all patients were in the acute phase (fever ≥ 3 days), the exact illness day at sampling (e.g., Day 4 vs. Day 6) could not be reliably extracted from the medical records. Platelet parameters may vary within the acute phase, and this variability may have influenced the results. Future prospective studies should standardize blood sampling to a specific illness day to minimize this confounding effect. This study did not perform predictive performance analysis, such as receiver operating characteristic (ROC) curves or validation of cut-off values. Therefore, no clinical implementation or treatment guidance can be recommended based on these findings alone. Future studies should include such analyses to assess the predictive utility of platelet parameters for coronary artery lesions. Finally, due to incomplete medical records, we were unable to adjust for several potential confounders, including albumin, sodium, liver enzymes, aspirin use, and illness day at blood sampling. Although we adjusted for CRP, WBC, fever duration, and IVIG-related variables, residual confounding from these missing variables cannot be ruled out. Future prospective studies should address these limitations. The sample size of this study, particularly the number of patients with coronary artery lesions (*n* = 28), is relatively small. With 12 variables included in the multivariate logistic regression model, the event-to-variable ratio was approximately 2.3, which is below the recommended minimum of 10 events per variable. This increases the risk of overfitting and may lead to unstable coefficient estimates and inflated effect sizes. Therefore, these findings should be interpreted with caution and considered hypothesis-generating rather than definitive. Future studies with larger sample sizes and independent validation cohorts are essential to confirm the observed associations. Future studies should involve larger sample sizes, multi-center prospective research, more comprehensive biomarker detection, and long-term follow-up data to further validate and refine the study conclusions.

In summary, this study, through a systematic analysis of the correlation between platelet parameters and coronary artery lesions in children with KD, not only validated the core role of platelet activation in the disease process but also provided new insights for clinical practice, including dynamic monitoring of platelet parameters for early risk stratification, precise therapeutic intervention, and prognosis assessment. These findings suggest that platelet parameters are associated with coronary artery lesions in children with KD. However, due to the retrospective design and lack of predictive performance analysis (e.g., ROC curves, sensitivity/specificity), we cannot recommend direct clinical implementation at this time. Future prospective studies with larger sample sizes and standardized outcome definitions are needed to confirm these associations and evaluate their potential for risk stratification. Given the limited sample size and the risk of overfitting, these findings should be interpreted as preliminary and exploratory. Validation in larger, prospective cohorts is required before any clinical application can be considered.

## Data Availability

The original contributions presented in the study are included in the article/Supplementary Material, further inquiries can be directed to the corresponding author.
